# The Multidisciplinary Management of Fused Maxillary Lateral Incisor with a Supernumerary Tooth in Cleft Lip Adolescence

**DOI:** 10.1155/2014/459416

**Published:** 2014-01-05

**Authors:** Ahmet Yagci, Kenan Cantekin, Suleyman Kutalmis Buyuk, Kansad Pala

**Affiliations:** ^1^Department of Orthodontics, Faculty of Dentistry, Erciyes University, 38039 Kayseri, Turkey; ^2^Department of Pediatric Dentistry, Faculty of Dentistry, Erciyes University, 38039 Kayseri, Turkey; ^3^Department of Restorative Dentistry, Faculty of Dentistry, Erciyes University, 38039 Kayseri, Turkey

## Abstract

Fusion, an uncommon anomaly of the hard dental tissues, is potentially the cause of clinical problems related to esthetics, tooth spacing, and other periodontal complications. This paper describes a multidisciplinary approach involving surgical, endodontic, restorative, and orthodontic attention for the successful, functional, and esthetic rehabilitation of a maxillary left lateral incisor fused with a supernumerary tooth in unilateral cleft lip adolescence in contralateral side. After clinical and radiographic examinations, a fusion between the left maxillary lateral incisor and a supernumerary tooth was diagnosed in the patient, and a small connection was detected between the pulp systems of the two root canals. The case reported in this paper presents the successful resolution of a fused maxillary lateral incisor with a supernumerary tooth, using endodontic, surgical, restorative, and orthodontic management. The decision made in extracting or retaining the fused tooth depends on the arch discrepancy and esthetic needs. Future studies, with long-term followup, will be helpful in evaluating the long-term efficacy of the different treatment options.

## 1. Introduction

The excessive mesiodistal width of a clinical crown might indicate the presence of a double tooth. The term double tooth is often used to describe fusion or germination, both of which are primary developmental abnormalities of the teeth that may require treatment for esthetic, orthodontic, and functional reasons [1]. Fusion occurs due to the union of two or more, separately developing, tooth buds at dentinal level, presenting one single large tooth structure and an increase in tooth number of the affected dentition, especially when it takes place between normal and supernumerary teeth [2]. The etiology of fusion is uncertain, with trauma, diseases, or genetics having been suggested as possible causes [3]. Clinically, fused teeth appear as a broad crown with a vertical groove extending toward the gingival sulcus. The pulp chamber and the root canals can either be joined or separate [4].

Fusion is an uncommon anomaly of the hard dental tissues, potentially causing clinical problems related to esthetics, tooth spacing, and other periodontal difficulties. The incidence of fusion is approximately 0.1% in permanent dentition and 0.5% in primary dentition, for the Caucasian population [5].

This case report describes the successful, functional, and esthetic rehabilitation of a maxillary lateral incisor fused with a supernumerary tooth in unilateral cleft lip adolescence. The adopted multidisciplinary approach considered surgical, endodontic, restorative, and orthodontic aspects of the treatment.

## 2. Case Report

**Figure 1:**
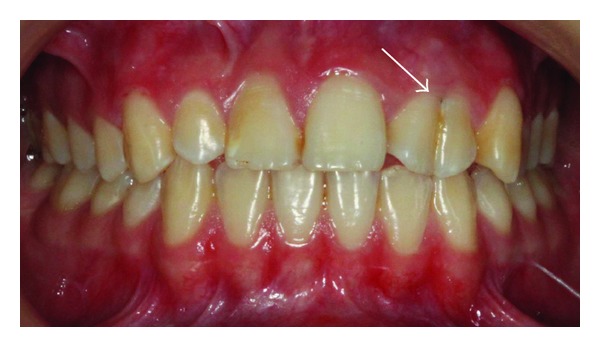
Clinical appearance of fused teeth; labial intraoral view.

**Figure 2:**
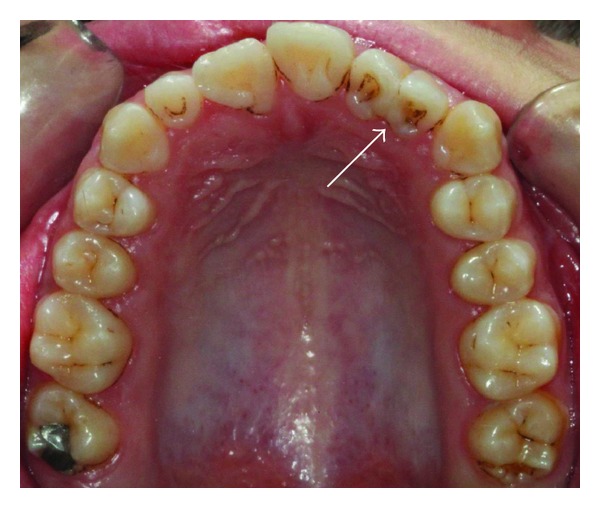
Clinical appearance of fused tooth; intraoral palatinal view.

**Figure 3:**
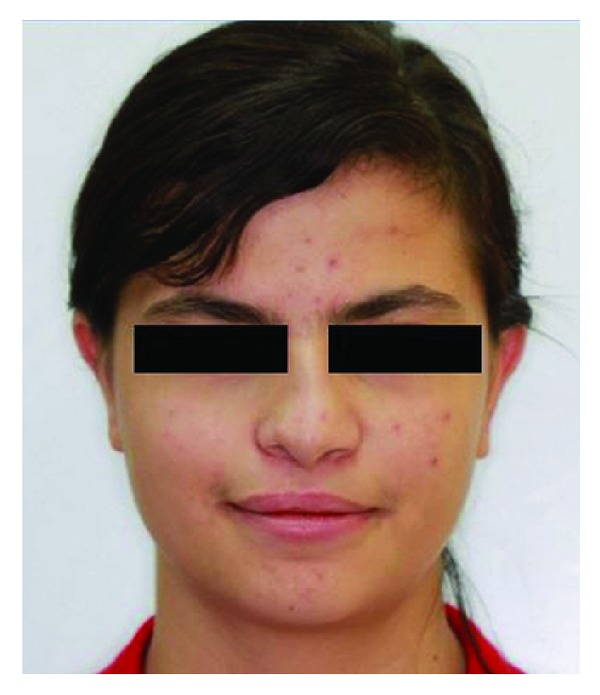
Extraoral front view of patient.

**Figure 4:**
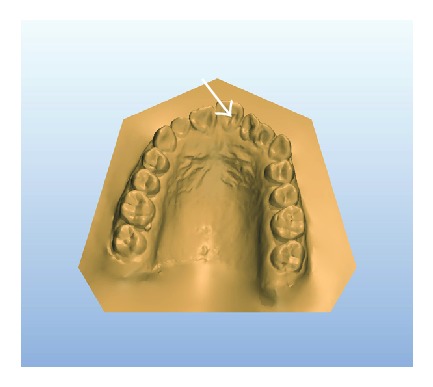
Preoperative 3D model: occlusal view showing mild anterior crowding with maxillary anterior teeth and Bolton's discrepancies.

**Figure 5:**
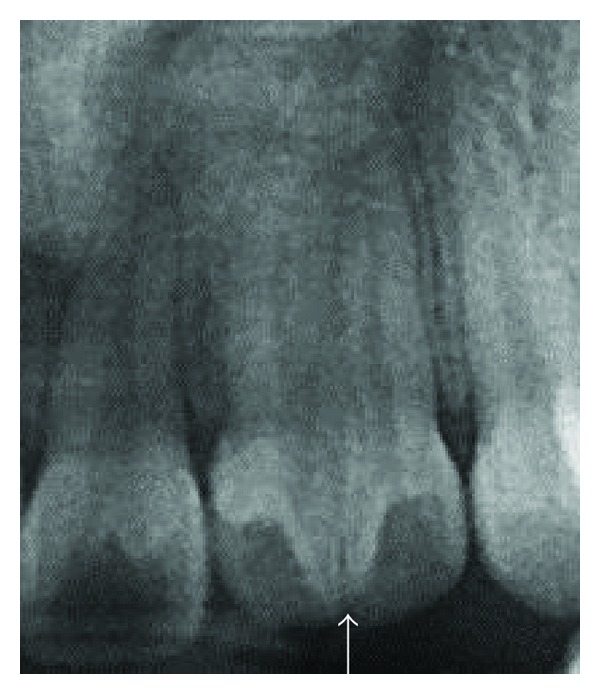
Preoperative radiograph revealing midroot connections between the root canals.

A 14-year-old female patient was introduced to the Department of Pediatric Dentistry, with the maxillary left lateral incisor exhibiting abnormal crown morphology. The tooth was wider mesiodistally (Figures 1–4), and a groove was observable between the clinical crowns. After clinical and radiographic examinations, a fusion between this maxillary lateral incisor and a supernumerary tooth was diagnosed and small connection was detected between the pulp systems of the two root canals (Figure 5). Extraoral examination of the hard and soft tissues revealed the presence of a unilateral lip cleft (Figure 3). Past medical history indicated that the cleft lip was closed when she was 12 months old. There had been no significant trauma history during pregnancy. Thermal pulp testing gave a normal response; there was no tenderness to percussion, and probing revealed no periodontal pocketing around the tooth. Lateral cephalometric radiographs revealed a skeletal class I (ANB = 2.5°) relationship with a normal plane angle (Sn-GoGn = 32.3°). The molar relations were class I relations on both the left and right sides. There was some anterior crowding in the upper arch and a midline shift according to the right central incisor (Figures 1, 2, and 4).

The proposed treatment plan was explained to the patient and her family. With their permission, the tooth was anaesthetized and isolated for endodontic treatment. Endodontic access cavities were prepared, on the mesial and distal sides of the palatal surface, using a number 2 round bur and an EX 24 bur (nonend cutting tapered fissure; Mani Inc., Tochigi, Japan). Pulp extirpation was performed using a barbed broach (Dentsply Maillefer, Ballaigues, Switzerland) and K-files (Mani Inc.). The canals were thoroughly debrided via copious irrigation with sodium hypochlorite (2.5%), followed by saline (0.9%). Coronal flaring of the root canals was done by using Gates-Glidden drills numbers 1 to 4 (Mani Inc.). The working lengths were determined by using an apex locator (Propex; Dentsply Maillefer) and confirmed radiographically. Cleaning and shaping of the root canal systems was completed using a step-back technique (apical enlargement was carried out up to ISO number 40). Again, the canals were liberally irrigated with sodium hypochlorite (2.5%), followed by saline (0.9%). The master apical file in both canals was ISO size 40. The roots were obturated using the lateral condensation technique with gutta-percha and AH-26 as sealers (Dentsplay, Konstanz, Germany). The mesial and distal access cavities were then sealed with resin composite and with zinc phosphate cement, respectively.

**Figure 6:**
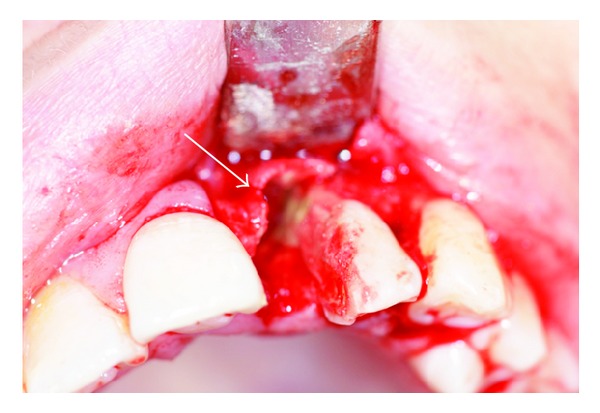
Removing of the mesial part.

**Figure 7:**
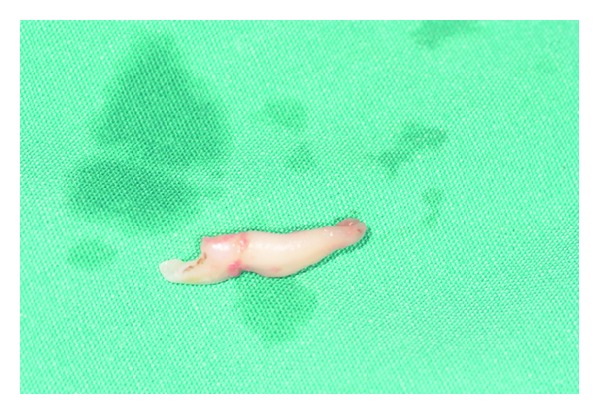
Mesial part of the fused teeth.

**Figure 8:**
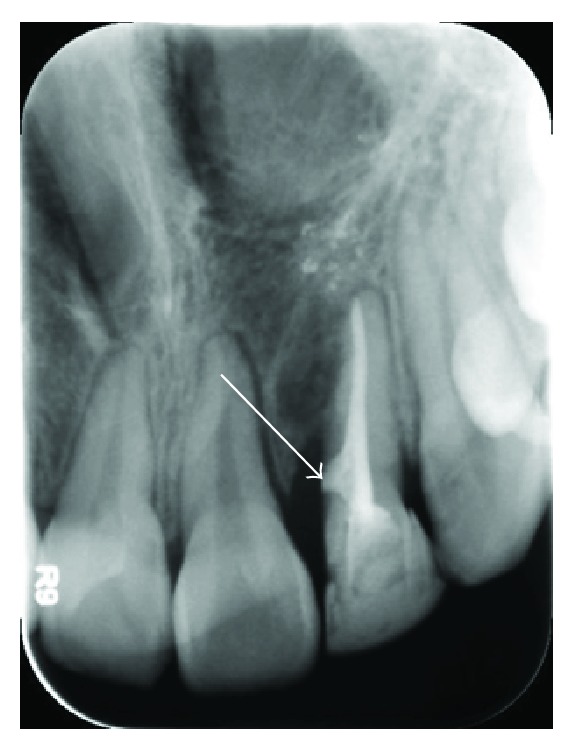
Postoperative radiograph: the defect left by the extraction of the mesial tooth is sealed with MTA.

After examining the outline and position of the roots, it was decided to remove the supernumerary tooth. The crown was divided with a diamond bur. After the root canal treatment and hemisection, the supernumerary tooth was removed and the communicating mesial surface was filled with white mineral trioxide aggregate (ProRoot MTA, Dentsply, Surrey, UK) (Figures 6, 7, and 8). The tooth was then restored with composite resin (Z100, 3M Espe, CA, USA) and the patient was told to brush her teeth carefully, after every meal and to use dental floss to avoid accumulation of plaque. The patient reported no postoperative symptoms.

**Figure 9:**
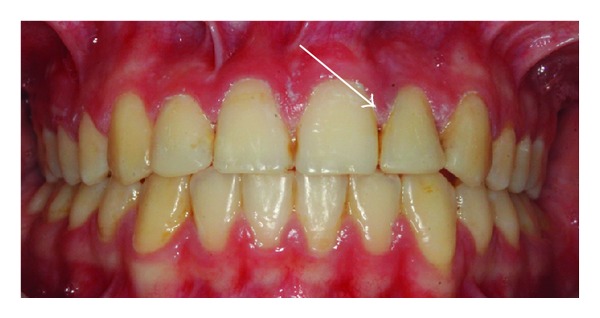
After orthodontic and restorative treatment clinical appearance of fused tooth; intraoral labial view.

**Figure 10:**
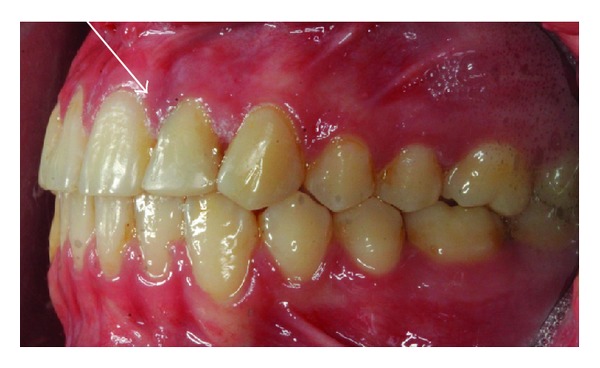
After orthodontic and restorative treatment clinical appearance of fused tooth; intraoral left labial view.

**Figure 11:**
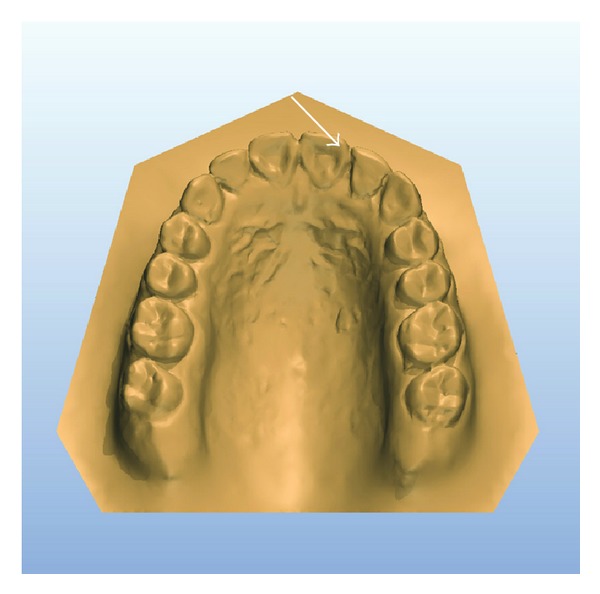
After treatment 3-dimensional model palatinal view.

The diastema on the left side, between the lateral incisor and the canine, was closed through orthodontic treatment. The upper midline shift was also corrected. At the end of the orthodontic treatment, composite build up was completed for the upper lateral incisors to compensate for Bolton's discrepancies (Figures 9–11). In this case, 0.022-inch slot MBT brackets were bonded. Tooth leveling and alignment procedures were accomplished with 0.016 nickel titanium wires (3 M Unitek, Monrovia, CA, USA), followed by rectangular nickel titanium wires. Space closure was done with rectangular stainless steel wire, and then normal finishing procedures were followed and the appliances were removed after 15 months of active treatment. A pleasing improvement in the dental occlusion was obtained. Normal retention was provided using a lower bonded retainer placed on the canines (Figures 9 and 10).

The patient was reexamined after six months. The maxillary lateral incisor was asymptomatic. Probing revealed no periodontal pocketing around the tooth and attachment was within normal biological limits. However, the patient's oral hygiene was inadequate in spite of the warnings given.

## 3. Discussion

Cleft lip (CL), with or without a cleft palate, is the most common congenital defect, with a prevalence varying from 1 in 500 to 1 in 2,500 live births, depending on the geographic origin and ethnic background of the individual [6–12]. Dental anomalies, including the number, position, morphology, structure, and eruption pattern, more frequently affect the primary and permanent dentition of children with CL [5, 12–19]. Dental anomalies are not exclusive to the cleft area, however, and can be present in no-cleft segments of the dental arch [3, 4, 12, 13]. Anterior teeth, especially the lateral incisors, are reported to be the most frequently absent, malformed, or fused with supernumerary teeth in CL cases [3, 12, 19–22].

Usually, fused teeth are asymptomatic and do not require treatment, and, if esthetically acceptable, the patient might even decide to retain the anomalous tooth [23]. However, double teeth can cause both esthetic and functional problems, including carious lesions in the grooves, particularly in the fusion zone involving asymmetries when fusion occurs in the anterior segment and malocclusions, especially when supernumeraries are involved [24]. The morphology of fused teeth varies, and complex forms with separated or fused (meeting in the radicular area) coronal pulp chambers are possible [23].

In this report, the endodontic, surgical, restorative, and orthodontic treatments, of a unilateral fusion of maxillary lateral incisor, are presented. As the etiological factors contributing to this anomaly have been identified, the fusion was thought to be related to a CL. The CL, which was located on the contra-lateral side of the fused teeth and was reported to have been closed at 12 months of age, might have caused this developmental anomaly. The most important challenges in fixing fused teeth are the mid-root connections between the root canals and the impact of fusion. Peyrano and Zmener [25] have reported that endodontic treatment is necessary when there are connections between root canals. The prognosis of maintaining pulp vitality, without root treatment, is declared to be poor in such cases [1, 25]. In cases like this, there is a risk of pulpal infection, which might develop from the periodontium or the connections between the root canals, because of poor oral hygiene. Because of this, the tooth was treated endodontically, and the mesial part of the tooth was sectioned and extracted. In addition, MTA was used to seal the window in the mesial surface after the extraction.

The case reported in this paper presents the successful resolution of a fused maxillary lateral incisor with a supernumerary tooth, using endodontic, surgical, restorative, and orthodontic management. The decision made in extracting or retaining the fused tooth depends on the arch discrepancy and esthetic needs. Future studies, with long-term followup, will be helpful in evaluating the long-term efficacy of the different treatment options.
